# Global Echoes: reflection on the use of interpretation in consultations in the UK

**DOI:** 10.1192/bji.2018.10

**Published:** 2019-05

**Authors:** Jonathan Cunliffe

**Affiliations:** Academic FY2, University of Sheffield, Sheffield Teaching Hospitals, UK. Email: jcunliffe@doctors.org.uk

**Keywords:** Community mental health teams, consent and capacity, education and training, out-patient treatment, post-traumatic stress disorder, psychotic disorders, social deprivation, transcultural psychiatry

## Abstract

Working as a foundation doctor in psychiatry I quickly noted the value of two things: the development of a strong therapeutic relationship and the importance of a detailed history. These two things are made much harder when your patient does not speak English and you do not speak their language. I present a reflection on my experiences with two patients who did not speak English, the role of interpreters and some common pitfalls when working with them.

*Barbarus hic ego sum, qui non intellegor ulli* (In this place I am a barbarian, because men do not understand me; Ovid, n.d.)

In exile from Ancient Rome, Ovid recognised the difficulty of not being understood. Without the means to explain himself, he was a barbarian: reduced to gestures and nodding his head, taunted by the locals, separated from civilisation. He was lost and alone.

For my patient, Abdul, it was not the perils of Ancient Rome but of modern day Somalia that plagued him. Abdul had experienced horrible trauma at home and fled to the UK, where he was relocated to Sheffield and housed in a small flat above some shops in the city centre. It was round the back of these shops that I stood waiting for an interpreter who would never arrive, ready to speak to Abdul about his post-traumatic stress disorder. Eventually accepting that I was on my own, I went upstairs and introduced myself to Abdul, not wanting to leave him rejected without saying hello. We sat silently in his bedroom, waiting for the possibility of company, making small talk with our faces. Abdul described the contents of his bedside table to me, in an incomprehensible way, and I replied with incomprehensible apologies. After a few minutes, we accepted defeat and parted, amicably, but both with a degree of frustration at our inability to understand one another.

As psychiatrists, we act as interpreters every day, translating a patient's internal reality into something tangible, which we attempt to make sense of. But in Abdul's case it wasn't greater clinical acumen or a stronger therapeutic relationship I was in need of; it was a real interpreter, one who could speak his language.

Working as a foundation doctor in psychiatry, I quickly picked up the importance of two things: first, how the development of a strong therapeutic relationship meant patients could trust you, would disclose things to you and might listen to you; and, second, that a detailed history is vital to understanding what symptoms a patient is experiencing and exploring the nuances of those symptoms. The second flows from the first, and both are key to making an accurate diagnosis and developing a treatment plan that can help your patient. Both of these things are made much harder when your patient does not speak English and you do not speak their language.

Limited language proficiency has been shown to be associated with fewer mental health care visits (Ohtani *et al*, 2015), and assessment in a patient's non-primary language can lead to incomplete or distorted histories (Bauer & Alegría, 2010). A lack of proper interpretation could even be deemed unethical, with issues raised around consent, confidentiality and the ability to make a good clinical decision (Blake, 2003). These issues are particularly relevant when using an often-tempting informal interpreter, such as a close family member. Hence, it seems wholly appropriate for the Royal College of Psychiatrists to recommend the use of professional interpreters (The Royal College of Psychiatrists, 2013); however, these can be hard to come by, and even with a trained interpreter there can be difficulties.

Fatima was psychotic. She described being abused in Pakistan and could now see figures from her past dancing outside the window. This was explained to me through our interpreter, Mina, with whom I met Fatima on three consecutive occasions; over these sessions I got to know both Fatima and Mina, the consistency a real blessing in building a working relationship between the three of us. However, our relationship was not without problems. At medical school, you are taught that when working with an interpreter you should speak to and look at your patient, not the interpreter, in order to build a relationship through your non-verbal behaviour and pick up clues from the patient's, but outside the classroom this is hard to do! I found myself drawn to Mina, her giving me the answers to my questions and me giving her eye contact in return, and had to remind myself that I was speaking to Fatima, rather than having a conversation about her with someone else.

Talking to Fatima helped me to focus on her behaviour, and I hope showed her I was interested in what she had to say, even if I did not understand the words coming from her lips. At times, I also had to question whether the words that reached my ears were the same ones that Fatima had spoken, with Mina occasionally coming across as a saboteur in our game of Chinese whispers. Long sentences would emerge as one or two words, while from grunts flowed rivers of meaning. I found it hard to decipher whether Mina was giving appropriate cultural context, or telling me what she thought I wanted to hear.

I'm not the first to experience these difficulties, and I doubt I'll be the last; Farooq and Fear explored the subject thoroughly, and highlight my problems with condensation, omission and addition (Farooq & Fear, 2003). They also offer some helpful tips for working with interpreters in the psychiatric setting: meet before, review after; speak slowly and clearly; and concentrate on non-verbal communication.

The difficulties described above relate to errors of direct interpretation, but it's also possible to miss the point through a lack of cultural context. I was fortunate when working with Fatima, as Mina came from a similar cultural background to her and so was able to set ideas and attitudes in context, but without that you can find yourself singing from a different hymn sheet to your patient (a phrase which itself may require context to translate!). Authors in this journal have highlighted the importance of cultural insight in articles about the mental health needs of and services for asylum seekers and refugees (Sen, 2016; Taylor-East *et al*, 2016). However, in a crisis setting, makeshift plans may mean that informal translation is more common, giving those most in need a lower standard of care.

Highly trained interpreters, and doctors who know how to work with them, can help reduce errors in translation and understanding, leading to improved care for some of the most vulnerable patients. When they find themselves lost and alone, we must offer someone who speaks their language. I was challenged by my experiences with both Abdul and Fatima to think about how to build a relationship without speaking, and also about the essential role of interpretation.

Identifying details in the cases above have been altered in order to maintain patient confidentiality.

## References

[ref1] Bauer A. M. & Alegría M. (2010) Impact of patient language proficiency and interpreter service use on the quality of psychiatric care: a systematic review. Psychiatric Services, 61(8), 765–73. 20675834 10.1176/appi.ps.61.8.765 PMC2946248

[ref2] Blake C. (2003) Ethical considerations in working with culturally diverse populations: the essential role of professional interpreters. CPA Bulletin de l'APC, 35(3), 21–23.

[ref3] Farooq S. & Fear C. (2003) Working through interpreters. Advances in Psychiatric Treatment, 9, 104–109.

[ref4] Ohtani A., Suzuki T., Takeuchi H., (2015) Language barriers and access to psychiatric care: a systematic review. Psychiatric Services, 66(8), 798–805. 25930043 10.1176/appi.ps.201400351

[ref5] Ovid, Ovid: Tristia V. Available at: http://www.thelatinlibrary.com/ovid/ovid.tristia5.shtml (accessed 18 February 2018).

[ref8] Royal College of Psychiatrists (2013) Vulnerable Patients, Safe Doctors: Good Practice in Our Clinical Relationships. RCPsych.

[ref6] Sen P. (2016). The mental health needs of asylum seekers and refugees – challenges and solutions. BJPsych International, 13(2), 30–32. 29093893 10.1192/s2056474000001069 PMC5619616

[ref7] Taylor-East R., Rossi A., Caruana J., (2016) The mental health services for detained asylum seekers in Malta. BJPsych International, 13(2), 32–35. 29093894 10.1192/s2056474000001070 PMC5619617

